# Health Care System Distrust, Race, and Surrogate Decision-Making Regarding Code Status

**DOI:** 10.1089/heq.2022.0044

**Published:** 2022-10-27

**Authors:** Sang Yoon Na, James E. Slaven, Emily S. Burke, Alexia M. Torke

**Affiliations:** ^1^ Department of Medicine, Indiana University School of Medicine, Indianapolis, Indiana, USA.; ^2^ Department of Biostatistics and Health Data Science, Indiana University School of Medicine, Indianapolis, Indiana, USA.

**Keywords:** race, trust, decision-making, resuscitation

## Abstract

**Purpose:**

Previous studies have shown that black patients are more likely to prefer life-sustaining treatments such as cardiopulmonary resuscitation at end-of-life (EOL) compared to non-Hispanic white patients. Given prior racial disparities in health care, distrust has been proposed to explain these preferences. As many hospitalized older adults require surrogates to make medical decisions, we explored surrogates' code status preferences and the role of trust in these decisions.

**Methods:**

We conducted secondary analyses of an observational study of patient/surrogate dyads admitted to three hospitals in a Midwest metropolitan area. Distrust was assessed using the Revised Health Care System Distrust Scale. A single item asked the surrogate which code status they thought was best for the patient, full code or do not resuscitate.

**Results:**

We enrolled 350 patient/surrogate dyads (101 black; 249 white). In bivariate analysis, higher proportion of black surrogates preferred full code (62.4% vs. 38.3%, *p*=0.0001). After adjusting for trust and sociodemographic and psychological covariates, race was still significantly associated with preference for full code (adjusted odds ratio=2.13; 95% confidence interval: 1.16–3.92; *p*=0.0153). Surrogate race was not associated with distrust in bivariate or multivariable analysis, adjusting for sociodemographic and psychological covariates (*p*=0.3049).

**Conclusion:**

Although black race was associated with preferences for full code status, we observed no association between race and distrust. Differences in code status preference may be due to other factors related to race and culture. To ensure that patients are receiving EOL care that is consistent with their values, more work is needed to understand the cultural complexities behind EOL care preferences.

## Introduction

There is evidence that African American/black patients are more likely than white patients to prefer aggressive or life-sustaining treatments such as code status, ventilators, or dialysis at the end-of-life (EOL) even when chances of survival are low.^1–15^ These differences in EOL preference may be shaped through cultural perspectives and life experiences.^16^ Therefore, it is important to look at factors that influence EOL decision-making so that health care providers can better understand their patients and ensure that they offer care that aligns with patients' preferences. This is especially important because despite these differences, studies show that a majority of black individuals express a desire for a peaceful EOL, incorporating their spirituality and surrounded by family and friends.^17^

One factor that has been proposed to affect EOL treatment decisions is distrust in the health care system.^1,5^ Patients or family members with lower trust may be unwilling to accept limitations on medical treatments, fearing that they are based on issues such as cost or race rather than on the patient's welfare. While some studies have found greater distrust in the health care system by black persons compared to the non-Hispanic white population,^18–20^ most studies have not found that distrust affects preferences for medical treatments.^18–21^

Many hospitalized elderly patients have experienced cognitive decline resulting in a decreased capacity for decision-making. As such, these patients share partial or even all decisions, including those pertaining to EOL, with a surrogate.^22^ Surrogate decision-making differs ethically and emotionally from patient decision-making.^23^ Surrogates are encouraged to rely on the patient's own preferences regarding medical care, although these are often unknown. Surrogates often struggle with the burden of making life and death decisions for another as well as the potential for grief if the patient dies.^24^ Since surrogates play an integral role in patient EOL care, it is essential to look at factors that may influence their decision-making such as distrust in the health care system.^25^

We hope to address a gap in the literature by examining relationships between trust, surrogate decision-making, and race in a secondary analysis of an observational study of surrogate decision-making for older adults with cognitive impairment and dementia. We studied decisions regarding code status because it is one important and common component of EOL preferences. It is important because it involves highly invasive procedures during the resuscitation, and any patient who survives requires ongoing critical care. In contrast, a do not resuscitate (DNR) order can provide a very different EOL experience for the patient and the surrogate. Although the decision-making that goes into a code status decision may be complex, high-quality code status orders must be clearly documented as a single medical order, so they can be quickly acted upon in an emergency, and asking about code status is a routine part of hospital admission.

Our study hypotheses are that black surrogates show higher distrust of the health care system when adjusting for sociodemographic covariates and that distrust mediates the relationship between race and preference for full code status. We rely on an understanding of race as a social construct and racial differences as stemming from cultural and social factors, rather than biological racial differences.^26,27^ Such differences are often due to historical and present inequities in health care that may affect medical decision-making regarding life-sustaining treatment. We will explore possible explanations for differences in EOL preferences with the hope of reducing barriers to hospice use, improving delivery of care that patients desire, and providing more culturally sensitive health services.

## Methods

The present study is a secondary analysis of data collected to validate a survey of clinician/surrogate communication for older, hospitalized adults.^28,29^ The Indiana University Institutional Review Board approved the study.

### Setting and participants

The setting included three hospitals in a single Midwest metropolitan area: a university tertiary referral hospital, an urban safety-net hospital, and a suburban community hospital affiliated with the university. We enrolled patient/surrogate dyads. Patients were adults aged 65 years and older admitted to the internal medicine or medical ICU services of one of the three hospitals. We chose to focus on medical services because such patients often are admitted for unexpected illnesses, for which in-the-moment decision-making is common. Eligible surrogates had either been previously named as a health care representative by the patient or were the default surrogate under state law.

Although many patients have the involvement of more than one person, we asked the primary treating physician to identify the person who had authority under state law. If there was more than one, the attending identified the person with whom they had most communication. To be eligible, the surrogate had faced at least one of three types of decisions during the current hospital stay, regarding (1) life-sustaining therapy such as code status or use of a ventilator, (2) procedures or surgeries requiring informed consent, or (3) placement in a nursing home or other facility. Patients were excluded if they lacked a surrogate decision-maker, the surrogate could not complete surveys in English, or the surrogate was a state-appointed guardian.

### Measures

Race and other demographic information was collected from the surrogate during the enrollment interview. Distrust was measured using the Health System Distrust Scale, a validated, 9 item scale with a Cronbach's alpha of 0.83.^30^ The code status preference of the surrogate was assessed during the baseline interview with an item that provided a definition of cardiopulmonary resuscitation and asked:

“…Thinking of (Patient's) current situation, would you want the doctors to try to revive him/her?”

Additional measures were included in this analysis because of their effects on preferences for code status based on prior literature.^31^ Communication quality was assessed using the Family Inpatient Communication Survey (FICS) (http://medicine.iupui.edu/IUCAR/research/tools/FICS).^29^ The FICS is a 30-item scale completed by the surrogate decision-maker to assess their perception of communication quality. It includes two subscales, information, and emotional support previously validated through factor analysis. Financial strain was assessed by an item addressing whether the participant judged the income as “comfortable,” “just enough to make ends meet,” or “NOT enough to make ends meet.” This item was used in further analyses due to high nonresponse to an item about income amount in the current study.

Overall psychological distress at baseline was assessed with the Kessler six-item (K6) Psychological Distress Scale.^32^ Anxiety was assessed with the Generalized Anxiety Disorder-7 (GAD-7), and depression was assessed with the Patient Health Questionnaire-9 (PHQ-9).^33^ Health literacy was measured by Rapid Estimate of Adult Literacy in Medicine (REALM)-Short Form and answers were dichotomized into greater than and less than sixth grade reading level.^34^

Death at the time of the 6–8-week follow-up interview was determined by surrogate report and chart review. Because belief in miracles has been found to be associated with EOL preferences for surrogates, we also asked an investigator-developed item regarding whether they believed that “divine intervention or a miracle could save the life” of the patient. Each patient's overall disease severity was measured via review of the electronic medical record (EMR) using the Cumulative Illness Rating Scale (CIRS), which assesses both number and severity of medication conditions and has been validated on hospitalized older adults.^35^ All previously published scales (FICS, PHQ-9, GAD-7 REALM, K6, and CIRS) have been validated in diverse populations.^28,30,32–37^

### Enrollment/administration

To identify eligible patients, research assistants (RAs) conducted daily (Monday–Friday) reviews of the EMR at each hospital. Patients were excluded for reasons, including lack of a family surrogate or evidence that the patient was making decisions independently. Among those with a potential need for a surrogate, the RA conducted a brief, 3–5-min screening telephone call with one of the patient's physicians (intern or resident in teaching setting and attending; or nurse practitioner or attending physician for nonteaching services) between days 2 and 4 of admission to a participating unit.

Physician report was used to determine whether the patient required a surrogate for all decisions and whether the surrogate had been faced with a decision in at least one of the predetermined categories. Informed consent and the surrogate interview were conducted in the hospital or by phone between hospital days 2 and 10. Surrogates consented for their own participation. Because the study involved patients who were entirely unable to make decisions, surrogate consent for the patient was obtained in all cases.

### Data analysis

Bivariate analyses were performed to determine how distrust and code status were associated with the demographics and clinical variables of interest. As distrust is a continuous measure, analysis of variance (ANOVA models) and regression analyses were performed when analyzing distrust, and chi-square and ANOVA models were performed when analyzing preference, a dichotomous outcome. Multivariable models were then performed, based on the significance of the bivariate models, using a cut-point of 0.20, as well as the experience of the clinical team, while also ensuring that there was no collinearity between the variables. Generalized Linear Models were performed, due to the capability of modeling both the continuous and dichotomous outcomes, using ANCOVA and logistic regression methods, with the addition of covariates. All analytic assumptions were verified, with log-transformations or nonparametric tests being performed where necessary. All analyses were performed using SAS v9.4 (SAS Institute, Cary, NC).

We tested whether distrust mediated the relationship between race and code status use standard sequential steps.^38^ We tested for associations between (1) race and code status, (2) race and distrust, and (3) the attenuating effect of distrust on the race/code status relationship.

## Results

### Patient and surrogate characteristics

We identified 799 eligible patient/surrogate dyads and enrolled 369 dyads. Five dyads withdrew, for a final enrollment of 364 dyads (45.6%). Two surrogates had incomplete responses for the measures included in this analysis and were excluded. Patients who identified as neither white nor black were excluded from the analysis (final sample 350 dyads). Patients had an average age of 81.82 (8.29) and were 27.9% black (Table 1). None of the black patients was Hispanic/Latino. There were two dyads where the patient was white and the surrogate was black.

**Table 1.** Patient and Surrogate Characteristics

Characteristics	Number (%)^a^
Patient (*n*=350)	
Age, mean (standard deviation)	81.81 (8.35)
Sex	
Female	215 (61.4)
Male	135 (38.6)
Race	
Black	99 (28.3)
White	251 (71.7)
Living situation	
Institution	124 (35.4)
Home with someone	177 (50.6)
Alone at home	49 (14.0)
Illness severity CIRS, mean (standard deviation)	24.40 (6.04)
Admission location	
Critical care/ICU	90 (25.9)
Hospitalist/floor	257 (74.1)
Reason for patient incapacity	
Dementia	212 (60.6)
Stroke	21 (6.0)
Delirium	20 (5.7)
Medication	23 (6.6)
Other	105 (30.0)
Surrogate (*n*=350)	
Age, mean (standard deviation)	58.34 (11.37)
Sex	
Female	247 (70.6)
Male	103 (29.4)
Race	
Black	101 (28.9)
White	249 (71.1)
Relationship to patient	
Spouse	61 (17.4)
Child	240 (68.6)
Other	49 (14.0)

^a^ Values are number (percent) unless otherwise indicated. Values are means (standard deviations) for linear continuous variables, medians (IQRs) for skewed continuous variables, and frequencies (percentages) for categorical variables. Some variables do not sum to 350 due to missing data.

CIRS, cumulative illness rating scale; IQR, interquartile range.

### Preference for full code

**FIG. 1.**
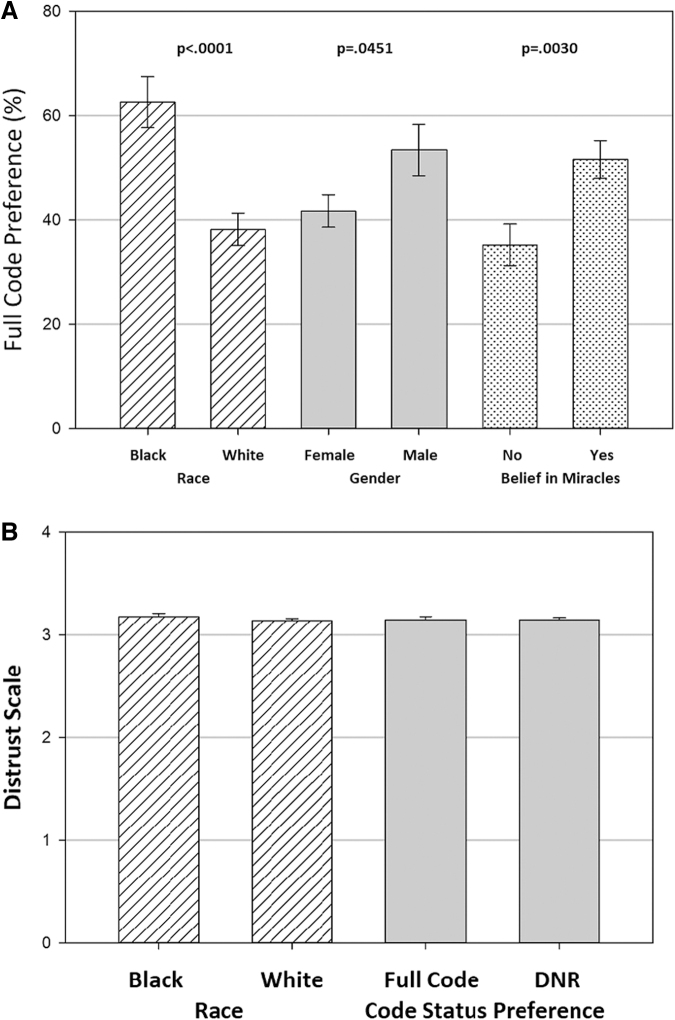
Percent of surrogates preferring full code status by race (shown in *striped bars*), gender (*gray bars*), and belief in miracles (*dotted bars*) **(A)** and distrust in the health system, by race (*dotted bars*) and code status (*gray bars*) preferences **(B)**.

In bivariate analysis, higher proportion of black surrogates showed preference for full code (62.4% vs. 38.3%, *p*=0.0001; Table 2 and Fig. 1). After adjusting for distrust and sociodemographic and psychological covariates, black race was still significantly associated with preference for full code (adjusted odds ratio [aOR]=2.13; 95% confidence interval [CI]: 1.16–3.93; *p*=0.0153).

**Table 2.** Association Between Preferences for Full Code Status and Other Variables

Variable	Full code (***n***=158)	Do not resuscitate (***n***=192)	***p*** (bivariate)	aOR (95% CI) (multivariate)
Distrust	3.15 (0.33)	3.15 (0.32)	0.9937	0.77 (0.35–1.71); *p*=0.5240
Race				
Black	62 (62.6)	37 (37.4)	<0.0001	Reference
White	96 (38.3)	155 (80.7)		2.13 (1.16–3.92); *p*=0.0153
Surrogate age	57.15 (11.60)	59.32 (11.12)	0.0777	0.99 (0.97–1.02); *p*=0.7210
Surrogate gender				
Female	103 (41.7)	144 (58.3)	0.0451	Reference
Male	55 (53.4)	48 (46.6)		0.43 (0.24–0.78); *p*=0.0055
Surrogate views decision-making				
Surrogate makes final decision	65 (42.5)	88 (57.5)	0.2493	
Shared	79 (49.4)	81 (50.6)		
Doctor makes final decision	13 (36.1)	23 (63.9)		
Surrogate visitation with patient				
Sees >weekly or lives with	116 (46.0)	136 (54.0)	0.6737	
Sees weekly or less	40 (43.5)	52 (56.5)		
Education level	12 (11, 12)	12 (10, 14)	0.3292	
Depression	3 (1, 7)	3 (1, 6.5)	0.6794	
Anxiety	2 (1, 5.5)	3 (1, 6)	0.4992	
Overall distress	1 (0, 5)	2 (0, 4)	0.0793	0.99 (0.91–1.07); *p*=0.7340
Surrogate frequency of communication with hospital staff				
A few times a week or less	19 (40.4)	28 (59.6)	0.7482	
Once a day	62 (44.6)	77 (55.4)		
More than once a day	76 (46.6)	87 (53.4)		
Communication quality: information	73.5 (66, 84)	75 (67.5, 84)	0.8074	
Communication quality: emotional support	49 (46, 55)	49 (47, 56.5)	0.5027	
Financial strain				
Comfortable	83 (42.4)	113 (57.7)	0.4248	
Just enough to make ends meet	57 (50.0)	57 (50.0)		
Not enough to make ends meet	17 (46.0)	20 (54.1)		
Health literacy				
Below or at sixth grade level	99 (42.5)	134 (57.5)	0.1592	
Above sixth grade level	59 (50.4)	58 (49.6)		0.67 (0.39–1.16); *p*=0.1509
Surrogate education	12 (11, 12)	12 (10, 14)	0.3292	
Surrogate self-reported health status				
Excellent/very good	64 (41.8)	89 (58.2)	0.2724	
Good/fair/poor	94 (47.7)	103 (52.3)		
Spiritual well-being	33 (29, 36)	32 (27.5, 35)	0.1729	1.03 (0.97–1.09); *p*=0.3775
Miracles				
Do not believe	50 (35.2)	92 (64.8)	0.0030	
Believe	99 (51.6)	93 (48.4)		1.79 (1.02–3.14); *p*=0.0424
Past experience with a family member in the				
No	22 (57.9)	16 (42.1)	0.0943	
Yes	136 (43.6)	176 (56.4)		0.81 (0.34–1.91); *p*=0.6240
Experience of caring for a family member who was dying				
No	67 (44.1)	85 (55.9)	0.9642	
Yes	71 (43.8)	91 (56.2)		
Surrogate been admitted overnight to any hospital				
No	28 (36.4)	45 (61.6)	0.2341	
Yes	130 (46.9)	147 (53.1)		
Illness severity	25 (21, 30)	24 (20, 28)	0.0170	1.05 (1.00–1.09); *p*=0.0505
Patient had previously discussed kind of medical wishes				
No	49 (53.9)	42 (46.2)	0.0481	
Yes	108 (41.9)	150 (58.1)		0.84 (0.46–1.54); *p*=0.5681
Had discussed these wishes with surrogate				
No	5 (50.0)	5 (50.0)	0.5946	
Yes	103 (41.5)	145 (58.5)		
Patient age	79.12 (7.97)	84.04 (8.00)	<0.0001	0.92 (0.89–0.96); *p*<0.0001
Patient gender				
Female	94 (43.8)	121 (56.3)	0.4999	
Male	64 (47.4)	71 (52.6)		1.27 (0.73–2.23); *p*=0.3950

Bivariate analysis result values are means (standard deviations) for linear continuous variables, medians (IQRs) for skewed continuous variables, and frequencies (percentages) for categorical variables, with *p*-values from ANOVA models (Kruskal-Wallis for skewed) and chi-square tests (Fisher's if cell counts are small), respectively. Some variables do not sum to 350 due to missing data. Adjusted model result values are odds ratios (95% confidence intervals) for preferring “full code” with *p*-values from multivariate logistic regression models.

ANOVA, analysis of variance; aOR, adjusted odds ratio; CI, confidence interval.

Gender, belief in miracles, greater illness severity, and younger patient age also showed significant associations in multivariable analyses. Male surrogates showed greater preference over female surrogates for full code (53.4% vs. 41.7%, *p*=0.0451) maintaining this significance after adjusting for covariates (aOR=0.43; 95% CI: 0.24–0.78). Surrogates that indicated a belief in miracles also showed a significant association in favor of full code (51.6% vs. 35.2%, *p*=0.0030) (aOR=1.79, 95% CI: 1.02–3.14). Younger patient age showed significant association (aOR=0.92, 95% CI 0.89–0.96; *p*<0.0001) with preference for full code.

### Distrust in health care system

Surrogate race did not show significant association with distrust in bivariate or multivariable analysis adjusting for sociodemographic and psychological covariates (*p*=0.2950; Table 3 and Fig. 1). However, increasing surrogate age showed significant association with increasing distrust (*p*=0.0341). Higher surrogate ratings of communication (information and emotional support) were associated with lower distrust (*p*=0.0049, *p*=0.0031) in bivariate but not multivariable analyses.

**Table 3.** Predictors of Surrogate Distrust of the Health Care System

	Bivariate	Multivariate
Surrogate race		
Black	3.18 (0.35)	3.18 (0.04)
White	3.14 (0.31)	3.14 (0.03)
*p*	0.2950	0.3049
Surrogate age	0.003 (0.002)	0.003 (0.002)
*p*	0.0341	0.0440
Surrogate gender		
Female	3.15 (0.31)	3.18 (0.03)
Male	3.15 (0.35)	3.15 (0.04)
*p*	0.8768	0.4577
Views on patient autonomy		
Surrogate decision	3.15 (0.35)	3.17 (0.03)
Shared decision	3.15 (0.30)	3.15 (0.03)
Doctor decision	3.16 (0.32)	3.16 (0.06)
*p*	0.9962	0.9551
PHQ9	0.0004 (0.003)	0.001 (0.005)
*p*	0.8928	0.8640
GAD7	−0.0003 (0.004)	−0.0004 (0.006)
*p*	0.9260	0.9484
Distress scale	0.0032 (0.004)	
*p*	0.4410	
Surrogate frequency of communication with hospital staff		
A few times a week or less	3.18 (0.40)	
Once a day	3.19 (0.31)	
More than once a day	3.12 (0.30)	
*p*	0.1373	
Communication quality (information)	−0.004 (0.001)	−0.003 (0.002)
*p*	0.0049	0.2103
Communication quality (emotional support)	−0.008 (0.003)	−0.005 (0.005)
*p*	0.0031	0.3068
Past experiences		
Surrogate admission to any hospital		
No	3.15 (0.30)	
Yes	3.15 (0.33)	
*p*	0.8400	
Surrogate income		
Comfortable	3.14 (0.34)	3.15 (0.03)
Just enough to make ends meet	3.18 (0.30)	3.18 (0.04)
Not enough to make ends meet	3.15 (0.34)	3.15 (0.03)
*p*	0.6840	0.7969
Surrogate health literacy		
≤6th grade	3.15 (0.33)	3.15 (0.03)
>6th grade	3.16 (0.31)	3.17 (0.04)
*p*	0.7407	0.6995
Surrogate education	−0.007 (0.005)	−0.005 (0.006)
*p*	0.2073	0.3891
Surrogate self-reported health status		
Excellent/very good	3.13 (0.29)	
Good/fair/poor	3.17 (0.35)	
*p*	0.2872	
Spiritual well-being	0.0001 (0.003)	
*p*	0.7609	
Miracles		
Do not believe (strongly disagree to neither)	3.13 (0.31)	
Believe (agree or strongly agree)	3.17 (0.34)	
*p*	0.2361	
Illness severity	−0.002 (0.003)	
*p*	0.5401	
Discussed kind of medical wishes they'd want?		
No	3.18 (0.33)	
Yes	3.14 (0.32)	
*p*	0.3830	
Discussed these wishes wit you (surrogate)?		
No	3.03 (0.36)	
Yes	3.15 (0.32)	
*p*	0.2683	
Patient age	−0.002 (0.002)	
*p*	0.3819	
Patient gender		
Female	3.16 (0.31)	
Male	3.14 (0.34)	
*p*	0.5139	

Bivariate analysis result values are means (standard deviations) for categorical variables and regression slope parameters (standard errors) for continuous variables, with *p*-values from ANOVA and regression analyses, respectively. Multivariate analysis results are means (standard errors) for categorical variables and regression slope parameters (standard errors), with *p*-values from analysis of covariance models.

Our initial hypothesis aimed to look at trust as a mediator to explain the positive correlation between race and EOL preferences. However, a key criterion that the predictor variable (race) be associated with the mediator (trust) was not found.^39^ Because race was not associated with distrust, there was no evidence that distrust mediated the relationship between race and code status preferences.

## Discussion

The current study of surrogate decision-makers found that black race is associated with greater preferences for full code status, but did not find an association between surrogate distrust in the health care system and preference regarding code status. This is consistent with prior studies that looked at patient preferences. Although distrust in the health care system has been proposed as a potential factor in decision regarding life-sustaining treatments such as code status or hospice among black patients, larger, quantitative studies have not borne this out.^1,5,19,20^

These studies have found mixed results regarding the association of patients' distrust in the health care system and race, but more consistently find that distrust cannot explain racial differences in preferences for life-sustaining interventions. The current study expands these findings to surrogates who are making decisions for the patient. Attributing a surrogate's preferences for code status to distrust of the health care system may distract from other important reasons for these preferences.^27^

Similar to the current literature about patient's own preferences, we did find that black surrogates showed greater preference for full code status.^20,21^ There may be other cultural or social factors that affect treatment decisions such as code status. For example, several qualitative studies found that black research participants felt that continued life support was consistent with their religious faith and another study found significant associations between race and religious beliefs that may conflict with the goals of hospice and palliative care.^15,21^

Religious beliefs such as the belief that pain and suffering are to be endured rather than avoided may be more common in the African American Christian tradition.^5^ The present study did find a positive relationship between preference for full code and belief in miracles. However, the association between race and code status preferences was significant even when controlling for belief in miracles, suggesting other factors are at play. The present study did not explore other faith or cultural beliefs.

Disparities in access to high-quality advance care planning may also play a role in decision-making.^27^ Studies have found that black patients report less shared decision-making in general and are less likely to be offered advance care planning.^27,40^ Although there was a trend toward higher surrogate preferences for DNR status when the patient had previously expressed wishes for care, this did not reach statistical significance and needs exploration in a larger study.

An important consideration in interpreting these results is the nature of the surrogate's role compared to a patient making their own decisions. A surrogate should ideally make decisions based on their presumed or previously discussed wishes of the patient.^23^ Therefore, the surrogate's views on the health care system may not influence their EOL preferences for the patient. In prior research with a racially diverse group of surrogate decision-makers, we found that surrogates considered both patient preferences and their own judgment of what was best, which may have taken into account the surrogate's own views on the health system.^41^

In addition, the majority of the surrogates interviewed were children of the patients. Due to differences in age and health status between surrogates and patients, they may have had very different interactions with the health care system with respect to quality and access to care. We were not able to assess the match between surrogate and physician perspectives in this study.

Limitations of this study should be noted, including that surrogates were not given further guidance to what individuals or entities are encompassed in the term health care system. An individual may regard the health care system as specific health care workers they have encountered in the past such as nurses, physicians, and so on. This may lead to a more intimate evaluation of trust through assessments of previous personal interactions with individuals within the hospital that were involved in their care. However, the health care system may also be perceived in a larger scope to include insurance companies, hospital systems, and clinical researchers.^39^ An evaluation of distrust may be quite different in this setting as an individual may consider systemic barriers that have impacted their or their loved one's care.

Another limitation of this study was that our population pool was limited to white and black surrogates in one U.S. metropolitan area and did not include participants who were unable to complete the study in English. A deeper evaluation of cultural differences may be explored with a more diverse population in future studies. More work is needed to understand EOL care preferences to ensure discrepancies in choices by race are in support of patient values and not the consequence of institutional barriers.

There are important implications of our findings for clinical care. Inequities in advance care planning education and facilitation must be addressed early the disease trajectory so that surrogates are prepared to make difficult decisions.^42^ Misattributing differences in code status preferences to trust can interfere with efforts to explore the surrogate's decision-making process and identify their true reasons for code status preferences.^27^ Such endeavors can be carried out by several members of the interdisciplinary team, including physicians, chaplains, and others. While building trust has many benefits for surrogates, it may not affect treatment preferences.

In conclusion, our study found an association between preferences for full code status and black race, but this was not mediated by distrust in the health system. Belief in miracles was associated with preferences for full code status, suggesting that religious or spiritual beliefs may play a role. Our findings have important implications for communication with black surrogates. Other factors such as disparities in the quality of medical care may be factors in preferring full code status.^5,11,27^

## Abbreviations Used

ANCOVA: analysis of covariance
ANOVA: analysis of variance
aOR: adjusted odds ratio
CI: confidence interval
CIRS: cumulative illness rating scale
DNR: do not resuscitate
EMR: electronic medical record
EOL: end-of-life
FICS: family inpatient communication survey
GAD-7: Generalized Anxiety Disorder-7
IQR: interquartile range
PHQ-9: Patient Health Questionnaire-9
RAs: research assistants
